# Influence of Several Compounds and Drugs on the Renal Uptake of Radiolabeled Affibody Molecules

**DOI:** 10.3390/molecules25112673

**Published:** 2020-06-09

**Authors:** Javad Garousi, Anzhelika Vorobyeva, Mohamed Altai

**Affiliations:** 1 Department of Immunology, Genetics and Pathology, Uppsala University, 751 85 Uppsala, Sweden; javad.garousi@igp.uu.se (J.G.); anzhelika.vorobyeva@igp.uu.se (A.V.); 2 Research Centrum for Oncotheranostics, Research School of Chemistry and Applied Biomedical Sciences, National Research Tomsk Polytechnic University, 634 050 Tomsk, Russia; 3 Division of Oncology and Pathology, Kamprad Lab, Department of Clinical Sciences, Lund University, 222 43 Lund, Sweden

**Keywords:** affibody molecules, radiolabel, ^99m^Tc, kidney, reabsorption, renal uptake

## Abstract

Affibody molecules are the most studied class of engineered scaffold proteins (ESPs) in radionuclide molecular imaging. Attempts to use affibody molecules directly labelled with radiometals for targeted radionuclide therapy were hampered by the high uptake and retention of radioactivity in kidneys. Several promising strategies have been implemented to circumvent this problem. Here, we investigated whether a pharmacological approach targeting different components of the reabsorption system could be used to lower the uptake of [^99m^Tc]Tc-Z_HER:2395_ affibody molecule in kidneys. Pre-injection of probenecid, furosemide, mannitol or colchicine had no influence on activity uptake in kidneys compared to the control group. Mice pre-injected with maleate and fructose had 33% and 51% reduction in the kidney-associated activity, respectively, compared to the control group. Autoradiography images showed that the accumulation of activity after [^99m^Tc]Tc-Z_HER2:2395_ injection was in the renal cortex and that both maleate and fructose could significantly reduce it. Results from this study demonstrate that pharmacological intervention with maleate and fructose was effective in reducing the kidney uptake of affibody molecules. A presumable mechanism is the disruption of ATP-mediated cellular uptake and endocytosis processes of affibody molecules by tubular cells.

## 1. Introduction

Advances in protein engineering such as molecular display techniques made it possible to generate alternatives to monoclonal antibodies (mAbs). A promising type of binding proteins is the non-immunoglobulin engineered scaffold-based affinity proteins (ESPs) [1,2]. Of these, affibody molecules have been studied the most for radionuclide-based molecular imaging [3]. Affibody molecules (7 kDa) are scaffold proteins with a size ca. 20-fold smaller than the size of intact mAbs. Affibody molecules binding with high affinity to several clinically relevant cancer-associated molecular targets (HER2, EGFR, HER3, IGF-1R, PDGFRβ and CAIX) have been developed [4,5]. In molecular imaging, radiolabelled affibody molecules proved to be advantageous in comparison with mAbs enabling higher sensitivity and specificity [5]. Due to their small size, affibody molecules accumulate rapidly in tumours, undergo rapid washout from non-targeted tissues, and have a short residence time in the circulation. It was shown in preclinical studies that affibody molecules provided at least 10-fold higher imaging contrast than monoclonal antibodies [4,6]. Clinical studies have recently demonstrated that injections of affibody molecules are well-tolerated by patients and do not elicit immune responses, which are key parameters in the development of any therapeutic protein-based agent [7]. Several preclinical studies have demonstrated that affibody molecules have the potential to target disease-specific antigens with a therapeutic intention [8,9]. Moreover, affibody structure permits site-specific conjugation of a payload for the development of targeted therapeutic agents with well-defined pharmacokinetics. We have recently demonstrated that affibody molecules could be successfully used as targeting vehicles for the delivery of a cytotoxic drug for cancer therapy [10].

A common issue for radiometal-labelled affibody molecules is the high radioactivity retention in kidneys. The size of affibody molecules (7 kDa) is below the kidney filtration barrier (60 kDa), making them readily filtered through the glomerulus [3,11]. After filtration, the affibody molecules carrying radionuclides are efficiently reabsorbed in proximal tubules of kidneys. If the label has residualizing properties, which is the case for most radiometals, the activity is retained in the tubular cells. This prevents the use of affibody molecules for targeted radionuclide therapy. Kidneys are radiosensitive organs and their exposure to high doses of activity used in therapy might result in renal damage and loss of function. There were several methods developed by other groups for reduction of renal uptake of radiolabelled proteins and peptides, such as infusion of basic amino acids or the gelatine-based plasma expander, Gelofusine. However, these methods were inefficient in the case of affibody molecules [11]. We have found that the renal reabsorption of anti-HER2 affibody molecules is mediated by an unknown scavenger receptor and that the amino acids comprising the HER2-binding site are involved in this process. The binding site is crucial for target recognition, and its re-engineering to reduce renal reabsorption of affibody molecules is practically impossible without compromising binding to the target.

Earlier, we succeeded in reducing the renal retention of radioactivity delivered by affibody molecules through the development of an innovative radiolabelling strategy that generated more “leaky” radiocatabolites escaping out of tubular cells. These efforts resulted in the development of anti-HER2 affibody molecule Z_HER2:V2_ labelled with the cytotoxic radionuclide rhenium-188 that could deliver high absorbed doses to tumours without exceeding kidney and bone marrow toxicity limits [12]. However, this kidney-preserving chemistry cannot be extended to other clinically relevant radiometals ^177^Lu, ^225^Ac and ^227^Th due to differences in chemical properties between rhenium and these radiometals.

An alternative strategy that permitted the use of affibody molecules for targeted radionuclide therapy was pretargeting. Pretargeting or multi-step targeting approach has been first applied to overcome the limitations associated with the use of directly radiolabelled mAbs in therapy [13]. We developed a novel pretargeting strategy based on affibody molecules and mediated by peptide nucleic acids (PNA) hybridization [14,15]. Using affibody-based PNA-mediated pretargeting, we obtained a 5-fold higher radioactivity accumulated dose to the tumour than to the kidneys. This approach provided doubling of median survival of ^177^Lu-pretargeted mice compared to control groups in preclinical setting [15].

Tolmachev et al. have developed a plasma half-life extension strategy based on coupling of affibody molecules to an albumin binding domain (ABD) aiming to increase the overall size and hydrodynamic radius of the affibody above the glomerular filtration barrier [16]. In vivo, fusion with ABD enabled a 25-fold reduction of renal uptake in comparison with the non-fused affibody molecule. Using this strategy for targeted delivery of ^177^Lu to HER2-expressing xenografts, they demonstrated that a single administration of ^177^Lu-CHX-A″-DTPA-ABD-(Z_HER2:342_)_2_ completely prevented the formation of tumours in mice.

The aforementioned strategies to reduce kidney uptake of affibody molecules are based on modulation of the physicochemical properties of the targeting moiety or properties of radionuclides and are developed for a given radiolabel-affibody combination. In this work, we intended to investigate whether a general pharmacological approach could be used for reduction of uptake of radiolabelled affibody molecules in kidneys. Such pharmacological approaches have been exploited earlier to reduce renal uptake of other peptide- and protein-based radiopharmaceuticals, such as somatostatin analogues [17,18,19] and designed ankyrin repeat proteins (DARPins) [20].

## 2. Results

**Figure 1:**
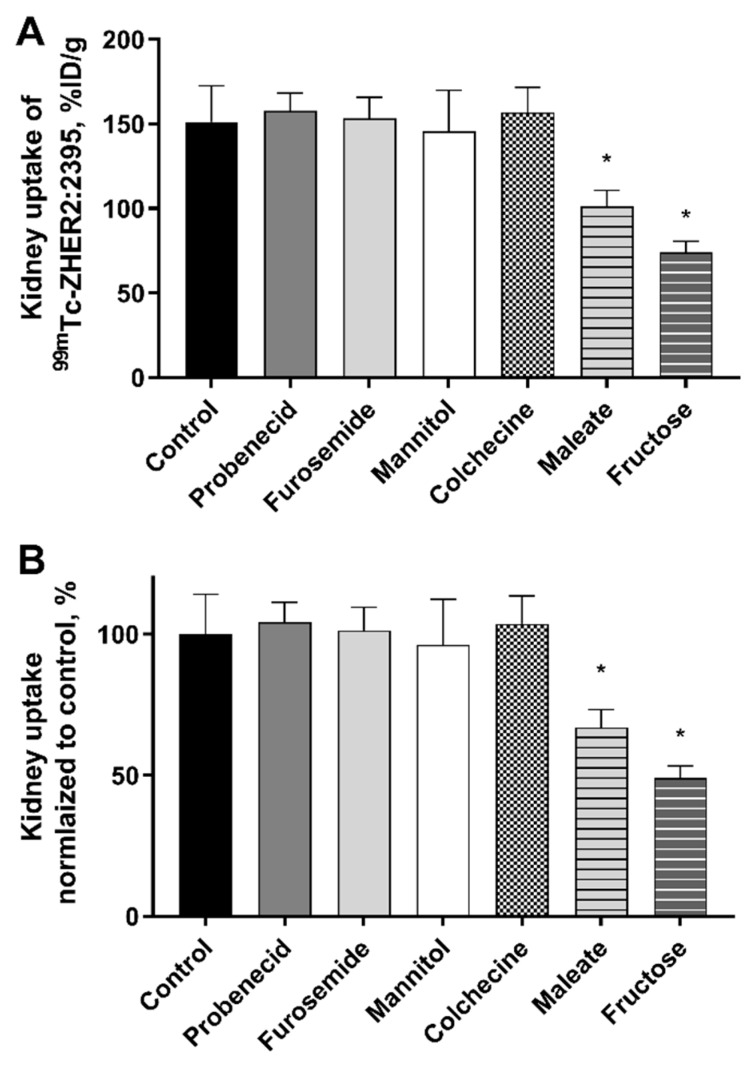
Kidney uptake of Z_HER2:2395_ affibody molecule labelled with ^99m^Tc in NMRI mice 4 h after injection. (**A**) The effect of various compounds on the kidney uptake of [^99m^Tc]Tc-Z_HER:2395_ represented as % ID/g. (**B**) The kidney uptake normalized to control in %. Data are expressed as an average of four animals ± SD. Asterisk (*) indicates a significant difference between control and the treated group (* *p* < 0.001, one-way ANOVA test).

Radiolabelling of Z_HER2:2395_-C affibody molecule with [^99m^Tc]Tc was performed in 96.5 ± 1.5% chemical yield and 100% radiochemical purity. Maleate, colchicine, probenecid, fructose, furosemide and mannitol act on various parts of the reabsorption system in kidneys and have been previously shown to interfere with the reabsorption of protein-based agents. To investigate their effect on reabsorption of affibody molecules in the kidneys, they were administered to healthy female NMRI mice prior to the injection of [^99m^Tc]Tc-Z_HER2:2395_ (Table 1). The control group received a single injection of [^99m^Tc]Tc-Z_HER2:2395._ The effect on biodistribution and kidney uptake was evaluated 4 h post [^99m^Tc]Tc-Z_HER:2395_ injection. Kidney uptake of activity in the maleate group was 1.5-fold (101.2 ± 9.6%ID/g) lower than in the control group (151.3 ± 21.3% ID/g) (Table 2 and Figure 1). A one-way ANOVA test did not reveal any significant differences (*p* > 0.05) in activity uptake in other organs or tissues. Fructose reduced the kidney uptake of [^99m^Tc]Tc-Z_HER2:2395_ by 2-fold (74.1 ± 6.4% ID/g) compared to the control group. However, an increased activity uptake was observed in the blood and other normal tissues (Table 2). No difference in the kidney uptake was observed in groups that received colchicine, furosemide, probenecid and mannitol compared to the control (Table 2 and Figure 1).

**Figure 2:**
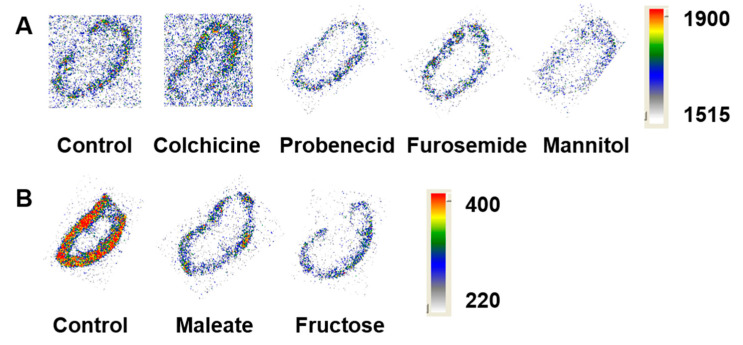
Representative ex vivo autoradiograms of kidney slices. NMRI mice were pre-injected with (**A**) colchicine, probenecid, furosemide, mannitol, (**B**) maleate and fructose prior to the injection of [^99m^Tc]Tc-Z_HER2:2395_. In the control groups mice were injected with [^99m^Tc]Tc-Z_HER:2395_ only and sacrificed 4 h post injection.

Autoradiograms of kidney sections of mice from the control and treated groups showed that the activity was mainly localized in the renal cortex for all studied groups (Figure 2). The level of activity in maleate and fructose treated groups was noticeably lower compared to the control (Figure 2B).

## 3. Discussion

ESPs and protein-based targeting agents below 60 kDa are readily reabsorbed in the renal tubular cells after glomerular filtration. Following reabsorption, lysosomal degradation of radiometal-labelled affibody molecules in the tubular cells generates “non-leaky”, residualizing radiocatabolites that are retained inside cells. This renders the radiosensitive kidney more prone to harmful radiation in targeted radionuclide therapy. Therefore, the use of affibody molecules for targeted radionuclide therapy is hampered by this elevated renal uptake of radioactivity. Direct pharmacological intervention using megalin blockers, Gelofusine and lysine, had no influence on the kidney uptake of affibody molecules [11]. Alternative strategies to reduce the renal accumulation of radioactivity observed with radiometal-labelled affibody molecules have resulted in several advancements [11,12,15,21,22,23,24,25,26,27]. In particular, the pretargeting and plasma half-life extension strategies demonstrated promising results in preclinical settings [15,16]. In this study, we extend on previous efforts by investigating whether the renal uptake of [^99m^Tc]Tc-Z_HER2:2395_ affibody molecule could be reduced by administration of other drugs and compounds that are known to act on different parts of the renal excretion pathway.

Colchicine is an anti-gout drug that interferes with the process of endocytosis mainly by inhibiting the microtubules polymerization and hence disrupting intracellular trafficking of organelles between different cell compartments [28]. Disruption of intracellular trafficking may interfere with the turnover/recycling of megalin scavenger receptor back to the luminal membrane. Rolleman et al. have shown that colchicine efficiently blocked the tubular uptake of the somatostatin analogue, [^111^In]In-DTPA-octreotide, in rat kidneys in a dose-dependent manner [17]. No effect of colchicine was observed with affibody molecules in the current study (Figure 1, Table 2). This lack of effect by colchicine was also observed for another class of ESPs, DARPins [20].

Next, we investigated if maleate would have any influence on the kidney uptake of affibody molecules. Maleate has been used in rats to induce an experimental model of Fanconi syndrome [29,30]. Maleate reduces cellular ATP by interfering with the citric acid cycle [31] and disrupts the energy-mediated endocytosis process. It was also proposed that maleate specifically inhibits membrane recycling of megalin during endocytosis [32]. However, Melis et al. have shown that megalin expression was not affected by maleate injections [19]. They have also demonstrated that the administration of sodium maleate reduced the renal uptake of [^111^In]In-DTPA-octreotide by 85% compared to the control group. An important finding was the less impressive reduction of renal uptake when maleate was given hours prior to the administration of the radiolabelled peptide, thus indicating a transient effect of maleate. Based on that, and in order to maximize the effect of maleate, we have chosen to administer maleate shortly before the injection of [^99m^Tc]Tc-Z_HER2:2395_ affibody molecule. Interestingly, we have observed a significant 33% decrease in the kidney activity uptake compared with the control group. It is worth mentioning that the convoluted segments of the proximal tubules, where reabsorption mainly takes place, are confined entirely to the renal cortex. Autoradiograms of kidney sections from the maleate group have demonstrated a reduction in the accumulated radioactivity in the cortex (Figure 2), which is in line with the biodistribution results.

Fructose is another compound that decreases intracellular ATP in liver and kidneys mainly through the sequestration of phosphates into metabolic intermediates [33]. Fructose injections in mice dramatically reduced tubular ATP in a dose-dependent manner. Doses of up to 20 mmol/kg fructose did not decrease the kidney uptake of [^111^In]In-DTPA-octreotide [17]. Mice in the current study were injected with 50 mmol/kg fructose immediately before the administration of [^99m^Tc]Tc-Z_HER2:2395_ affibody molecule. We observed a 2-fold decrease in the kidney-associated radioactivity compared with the control group. Interestingly, liver and blood activity levels were significantly elevated by fructose, but not by maleate (Table 2). We speculate that the higher accumulation of radioactivity in organs and blood in the presence of fructose could be due to reduced renal function imposed by the ketonic monosaccharide. The injected dose of fructose used in this study is close to the LD_50_ of the compound (80 mmol/kg), thus, it is of interest to see if lower doses or different administration schedules could provide a similar effect.

It was documented in the literature that the proximal tubules express various forms of organic transporters [34,35]. We tested if the anti-gout drug probenecid, known to inhibit organic anion transporters (OATs) and renal excretion of some drugs, would have any effect on the renal uptake of [^99m^Tc]Tc-Z_HER2:2395_. Earlier, Stahl et al. reported that probenecid administration reduced kidney uptake of [^111^In]In-DOTATOC by 30% [18]. However, administration of probenecid did not reduce the kidney uptake of affibody molecules (Figure 1 and Figure 2). This lack of probenecid effect was also observed for DARPins [20]. Together these data suggest that OATs do not play a significant role in the uptake of affibody molecules by the proximal tubular cells.

Furosemide, a loop diuretic, may affect the urinary and plasma concentrations of some co-administered drugs by either increasing the glomerular filter load or inducing an increased renal excretion in the tubules [36]. Mannitol is an osmotic diuretic which may induce forced diuresis, enhancing the elimination of certain drugs and toxins from the body. Both furosemide and mannitol had no effect on renal radioactivity accumulation post-[^99m^Tc]Tc-Z_HER2:2395_ injection. These results indicate that the capacity of reabsorption of small proteins, specifically affibody molecules, is neither dependent on the filter volume or load, nor on its rate of flow.

Although interesting, the effect of fructose and maleate on the kidney uptake of radiolabelled affibody molecules is suboptimal and hence cannot justify the use of these drugs in radionuclide therapy protocols involving radiolabelled affibody molecules. We believe that findings from this study may help in understanding the underlying mechanism of kidney uptake of affibody molecules, as well as be of help for researchers working with other classes of targeting agents with elevated kidney uptake. A similar approach may provide an indication for researchers whether they should optimize therapy protocols by co-administering blocking agents or adopt more efficient strategies, for example, pretargeting or plasma half-life extension. We, therefore, recommend not to generalize the findings from this study to other classes of targeting agents and that the kidney uptake lowering strategies should be evaluated for each class on a case-by-case basis.

## 4. Materials and Methods

Technetium pertechnetate ^99m^TcO_4_^−^ was provided by elution of ^99^Mo/^99m^Tc generator (Mallinckrodt-Tyco, Petten, The Netherlands) with sterile saline solution. Sodium maleate, D-fructose, colchicine, probenecid and L-lysine were purchased from Sigma (Sigma-Aldrich, Saint Luis, MO, USA). Furosemid (Takeda Pharma AB, Stockholm, Sweden), mannitol (Fresenius Kabi AB, Uppsala, Sweden), and Gelofusine (B. Braun Melsungen AG, Melsungen, Germany) were purchased as solutions for injections. Radioactivity was measured by an automated γ-spectrometer with a NaI(Tl) detector (1480 Wizard, Wallac, Turku, Finland).

### 4.1. Labelling of Z_HER2:2395_ with ^99m^Tc

Affibody molecules Z_HER2:2395_ were produced and purified as described by Ahlgren et al. [21]. Labelling was performed using a freeze-dried kit, containing 5 mg sodium α-d-glucoheptonate (Celsus Laboratories, Geel, Belgium), 75 µg SnCl_2_ (Fluka Chemika, Buchs, Switzerland) and 100 μg of EDTA (Sigma-Aldrich, Munich, Germany), as described earlier [37,38]. The lyophilized Z_HER2:2395_ (50 µg) was reconstituted in 50 µL degassed PBS and the solution was added to the content of one freeze-dried kit. Eluate containing ^99m^Tc (100 µL) was added. The mixture was incubated for 40 min at 95 °C. The conjugate was purified using a disposable NAP-5 size-exclusion column. To determine the radiochemical yield and radiochemical purity, iTLC-SG strips (Varian, Lake Forest, CA, USA) were eluted with PBS.

### 4.2. Compounds Acting on Tubular Reabsorption

A list of different compounds and drugs used in this study and their proposed mechanisms of action are shown in Table 1.

### 4.3. Animal Studies

All experiments had been approved by the Ethics Committee for Animal Research in Uppsala County (Permit Number: C4/ 2016) and were performed according to the Swedish laws on laboratory animal welfare. Thirty-two female NMRI mice, divided into eight groups of four mice, were used in the experiments. The average mouse weight was 27 ± 3 g, and the average kidney weight was 272 ± 35 mg at the start of the experiment. Mice and kidney weights did not differ significantly (*p* > 0.05, one-way ANOVA test) between the groups. For all experiments, 1 µg (60 kBq) of [^99m^Tc]Tc-Z_HER2:2395_ affibody molecule was injected intravenously into the tail vein (100 μL). In the treatment groups, mice were pre-injected with probenecid (20 mg/kg, 1 h, i.p.), furosemide (2.5 mg/kg, 5 min, i.v.), mannitol (400 mg/kg, 5 min, i.v.), colchicine (1 mg/kg, 5 h, i.p.), maleate (400 mg/kg, 5 min., i.v.) and fructose (50 mmol/kg. 5 min, i.p.) prior to the injection of [^99m^Tc]Tc-Z_HER2:2395_ affibody molecule. Control groups were injected with [^99m^Tc]Tc-Z_HER2:2395_ only. Animals were euthanized and dissected 4 h after injection of [^99m^Tc]Tc-Z_HER2:2395_ and organs were collected and measured for radioactivity using an automated gamma counter.

### 4.4. Autoradiography

Autoradiographic images of the kidneys from treated mice were acquired and compared with controls. After the completion of gamma counter measurements, two pairs of kidneys were taken from each group, and were then embedded in the OCT cryomedium, frozen at −80 °C, cut in 30 μm thick serial sections using a cryomicrotome, and thaw-mounted on glass slides. The slides with sections were exposed to phosphor screens overnight, scanned using a Cyclone Storage Phosphor System at a resolution of 600 dpi and analysed with OptiQuant image analysis software (Perkin Elmer, Waltham, MA, USA).

The study was done in two days. Day 1 included treatment groups and a control group, shown in Figure 2A, and day 2 included treatment groups and a control group shown in Figure 2B. The kidney sections represented in every panel were simultaneously exposed to one phosphor screen and the signal was acquired for the same period of time. The phosphor screens capture the activity and are scanned by the instrument’s laser to create a high-resolution digitized image with quantitative data. Each pixel in the image contains an intensity value. The colour range represents the range of intensity (unit per pixel) in the bitmap image. The range is determined by the minimum (Min) and maximum (Max) parameters and the colours can be adjusted manually. The relationship between intensity values in the image bitmap and colours was set to linear. The colours in the autoradiograms were adjusted to provide the maximal contrast to background.

### 4.5. Statistical Analysis

GraphPad Prism version 8.00 for Windows (GraphPad Software, San Diego, CA, USA) was used for statistical analysis. Biodistribution data were analysed using descriptive and dispersion statistics to measure; the frequency (percentage), distribution or central tendency (mean), dispersion or variation (standard deviation and one-way ANOVA test). The one-way ANOVA was evaluated with Bonferroni correction for multiple comparisons.

## Figures and Tables

**Table 1:** List of compounds administered before the injection of [^99m^Tc]Tc-Z_HER2:2395_ in Naval Medical Research Institute (NMRI) mice.

Compound	Suggested Mechanism of Action	Route of Administration and Time before Affibody Injection	Dose	Lethal Dose, 50% (LD_50_)
**Fructose**	Inhibits ATP-mediated endocytosis	i.p. t = −0.08 h	50 mmol/kg	83 mmol/kg (15 g/kg)
**Colchicine**	Inhibits recycling of megalin by disrupting microtubules	i.p. t = −5 h	1.2 mg/kg	1.6 mg/kg (i.p. mouse)
**Mannitol**	Diuretic, reduces contact time with scavenger receptors	i.v. t = −0.08 h	480 mg/kg	7470 mg/kg (i.v. mouse)
**Furosemide**	Diuretic, reduces contact time with scavenger receptors	i.v. t = −0.08 h	3 mg/kg	800 mg/kg
**Probenecid**	Reduces drug renal excretion by inhibiting organic anion transporter	i.p. t = −1 h	24 mg/kg	1000 mg/kg (i.p. mouse)
**Sodium maleate**	Inhibits ATP-mediated endocytosis	i.v. t = −0.08 h	400 mg/kg	3308 mg/kg (oral, rat) 600 mg/kg (i.p., rat)

**Table 2:** Biodistribution of [^99m^Tc]Tc-Z_HER2:2395_ in NMRI mice 4 h after injection alone (control) or after administration of compounds.

	**Blood**	**Salivary Glands**	**Liver**	**Spleen**	**GI Tract**	**Carcass**
**Control**	0.15 ± 0.06	0.37 ± 0.19	1.53 ± 0.59	0.36 ± 0.09	2.25 ± 0.49	2.93 ± 0.68
**Probenecid**	0.17 ± 0.02	0.36 ± 0.01	1.79 ± 0.21	0.51 ± 0.07	3.2 ± 1.53	3.34 ± 0.27
**Furosemide**	0.22 ± 0.04	0.46 ± 0.15	2.16 ± 0.16	0.57 ± 0.11	3.08 ± 0.77	3.97 ± 0.58
**Mannitol**	0.2 ± 0.04	0.48 ± 0.13	1.77 ± 0.35	0.47 ± 0.07	2.27 ± 0.42	3.42 ± 0.64
**Colchicine**	0.36 ± 0.04 *	0.89 ± 0.12 *	1.21 ± 0.27	1.16 ± 0.24 *	2.06 ± 0.51	5.69 ± 0.39 *
**Maleate**	0.17 ± 0.02	0.32 ± 0.04	2.02 ± 0.23	0.47 ± 0.03	3.37 ± 1.73	3.71 ± 0.38
**Fructose**	1.75 ± 0.13 *	1.2 ± 0.14 *	4.02 ± 0.62 *	2.07 ± 0.42 *	4.89 ± 0.67 *	21.5 ± 3.23 *

The activity in blood, salivary gland, liver and spleen is presented as % ID/g; for gastrointestinal (GI) tract and carcass is presented as %ID per whole sample. The values for the treated groups are presented as an average from four animals ± standard deviation (SD), for the control group—as an average of eight animals ± SD. Asterisk (*) indicates a significant difference between control and the treated group (* *p* < 0.01, one-way ANOVA test).

## Abbreviations

HER2	Human Epidermal growth factor type-2
ABD	Albumin Binding Domain
DARPins	Designed Ankyrin Repeat Proteins
ESPs	Engineered Scaffold-based affinity Proteins
mAbs	Monoclonal antibodies
OATs	Organic anion transporters
PNA	Peptide nucleic acids
i.p.	Intraperitoneal injection
i.v.	Intravenous injection
%ID/g	Uptake represented as percent of injected dose per gram of tissue
